# Cargo Cult Radiotherapy: The Illusion of Precision in Advanced Technologies

**DOI:** 10.7759/cureus.79005

**Published:** 2025-02-14

**Authors:** Susannah G Ellsworth, Christopher Wilke

**Affiliations:** 1 Radiation Oncology, University of Pittsburgh Medical Center, Pittsburgh, USA

**Keywords:** advanced radiotherapy, medical panglossianism, modern radiotherapy techniques, radiotherapy, technological applications in medicine

## Abstract

Emerging radiotherapy technologies such as proton therapy, MR-guided radiotherapy, and real-time adaptive radiotherapy share a common goal of improving radiotherapy outcomes by increasing the anatomic precision of treatment delivery. In this piece, we provide a critical view of “precision radiotherapy” by examining the assumptions underlying the theory and practice of these techniques. Our goal was not to provide an exhaustive review of published literature; rather, we strove to write an accessible and thought-provoking article that would challenge conventional wisdom and stimulate further discussion and debate.

## Editorial

Introduction

*“In the South Seas there is a Cargo Cult of people. During the war they saw airplanes land with lots of good materials, and they want the same thing to happen now. So they’ve arranged to make things like runways, to put fires along the sides of the runways, to make a wooden hut for a man to sit in, with two wooden pieces on his head like headphones and bars of bamboo sticking out like antennas-he’s the controller-and they wait for the airplanes to land. They’re doing everything right. The form is perfect. It looks exactly the way it looked before. But it doesn’t work. No airplanes land. So I call these things Cargo Cult Science, because they follow all the apparent precepts and forms of scientific investigation, but they’re missing something essential, because the planes don’t land.” *-Richard Feynman [1]

Nobel Prize-winning physicist Richard Feynman described the phenomenon of “cargo cult science”: going through the motions of a scientific endeavor without applying the requisite theoretical or intellectual rigor. Cargo cult methods superficially resemble scientific pursuits but fail to achieve meaningful results. In this paper, we argue that a fixation on anatomic precision in radiotherapy (RT) delivery represents a form of “cargo cult radiotherapy”: utilizing elaborate technological maneuvers to manipulate a system that remains incompletely understood. The result is a potentially harmful overemphasis on technical solutions to biological problems.

Great enthusiasm has surrounded technological progress in RT; modern advances in treatment delivery such as 3-D planning, intensity-modulated radiation therapy (IMRT), and stereotactic body radiation therapy (SBRT) represent not only technical milestones but also true progress in the fight against cancer. Advanced RT techniques now enable tumor ablation with minimal side effects in a wide variety of cancers. Many researchers have currently set their sights on leveraging emerging technologies such as proton/heavy ion therapy, real-time adaptive RT, and MR-guided RT to further improve outcomes, aided by considerable support from the medical device industry. These approaches (referred to throughout the manuscript as "precision RT") share a common goal of enhancing the physical precision of dose delivery, albeit by differing mechanisms.

Proponents argue that precision RT will substantially improve the therapeutic ratio of radiation by intensifying the dose in the tumor and decreasing the dose to neighboring organs at risk (OARs). This argument is directly rooted in the single most fundamental tenet of classical radiobiology: cell death (loss of proliferative capacity) increases with increasing the radiation dose in both tumor and normal tissue. The classical model predicts that increasing the dose to the tumor will increase cell kill and improve cure rates, while decreasing the dose to normal tissue will reduce toxicity. The natural extrapolation of this model is that stricter adherence to target coverage and OAR sparing will manifest as measurable improvements in disease control and normal tissue toxicity. Certainly, the predictions of classic radiobiological modeling appear to have been confirmed by successful tumor ablation with focal, dose-escalated radiation. Is it not then logical to continue pushing the limits of conformality and dose escalation, assuming consistent improvements in the continuum of treatment outcomes? In our view, however, conceptual errors undermine certain assumptions on which the precision RT paradigm is based, and practical considerations may limit the effective clinical utility of these techniques. The theory and practice of precision RT methods must, therefore, be thoroughly interrogated before these approaches are routinely integrated into clinical practice.

Technical solutions to biological problems: pernicious reification, the ecological fallacy, and medical Panglossianism

*“It just so happens that your friend here is only mostly dead. There’s a big difference between mostly dead and all dead. Mostly dead is slightly alive.” *-William Goldman, *The Princess Bride*

Medicine is an applied science, in which desired physiologic effects are elicited by the empiric implementation of abstract concepts. (That said, practice often presages theory; RT was used to shrink tumors decades before cancer was understood as a disease driven by aberrant DNA and radiation as a tool to destroy the deranged genetic material of the cancer cell.) Successfully translating abstract ideas to concrete applications, therefore, lies at the very heart of clinical care. As the psychologist John Dewey noted, “…abstraction…is the heart of thought; there is no way…to control and enrich concrete experience except through an intermediate flight of thought with conceptions, relations, abstracta" [2]. Reification is the cognitive mechanism by which an abstraction or concept is made concrete [3]. While reification is a neutral process, pernicious reification is “abstraction gone wrong, resulting in universalized, rigid and narrow [thinking]…it is to abstraction as disease is to health” [4]. Pernicious reification treats abstract models not as tools to aid in understanding some aspect of the real world but as reality themselves. Over time, clinical practice in radiation oncology has unintentionally fallen victim to a form of pernicious reification with respect to classical radiobiologic cell survival models. An understandable desire to standardize practice has resulted in a dogmatic approach to radiation dosing regimens, despite considerable uncertainty in how much dose is needed to cure an individual tumor and what safe dose limits are to neighboring normal tissues. RT approaches that overemphasize anatomic accuracy in dose delivery, thus, enshrine current practices in a sense of false certainty while threatening to crowd out potentially more creative, efficacious, and cost-effective approaches to improving outcomes in patients treated with radiation.

In the following section, we will consider the validity of the assumptions underlying RT paradigms that rely solely on increasing the anatomic accuracy of dose delivery, which can be roughly summarized as follows: 1) Local control increases with increased dose to the tumor. This assumption is directly informed by classical radiobiology and suggests that by enabling dose escalation and enforcing rigorous conformality of the correct prescription isodose line to the edge of the target, precision RT will improve disease control. 2) The optimal tumor dose is determined a priori by tumor histology and does not vary among individuals, within the tumor itself, or during treatment. 3) A decreased dose to normal tissue will reduce the incidence of side effects; toxicity risk is determined by dose-volume distribution and intrinsic tissue characteristics.

In classical radiobiology, cell survival curves for both tumor and normal tissue have a sigmoidal shape, reflecting laboratory experiments quantifying cell death following varying doses of radiation [5]. (In this context, cell “death” is considered equivalent to the loss of proliferative capacity.) These curves show that there is some dose below which no cell killing is detectable. At higher doses, there is an inflection point, after which cell kill is proportional to dose; once this sloped portion of the survival curve is reached, even small changes in dose yield observable decrements in cell survival. The survival curve flattens again once there is no additional cell killing with an additional dose. When this model is extrapolated to clinical RT courses, observable increases in local control are predicted to occur with dose-escalated RT while failing to achieve the prescribed dose within a target will result in local relapse: the so-called marginal miss. A final corollary is that a decreased dose to normal organs should reduce the incidence and severity of radiation-induced toxicity.

The predictions of this model, however, have not consistently been borne out in clinical practice. In a meta-analysis of randomized RT dose-escalation studies, Yamoah et al. found that, with only a few exceptions, dose escalation was not associated with either improved local control or overall survival [6]. Two related considerations can assist in understanding why dose escalation has not uniformly resulted in higher cure rates. First, while cell kill increases linearly with the radiation dose in vitro once the shoulder of the dose-response curve is overcome, tumor cure is only observed clinically once the entire reproductive potential of the tumor has been eliminated. While the degree of tumor cell killing may well be proportional to the dose, the observable clinical effect of cure or no cure is a binary endpoint; for example, there is no difference in the likelihood of cure with a 10% increase in the dose that proportionally increases cell kill from 80% to 88%.

Second, large interindividual variations in radiosensitivity, even among tumors of the same histology, mean that averaged cell survival curves for a given tumor type are not able to accurately predict what dose must be delivered to a *particular* tumor in order to destroy 100% of proliferative capacity. While it is trivial to predict that a dose of 70 Gy is more likely than 10 Gy to achieve a complete response in most cancers, it does not follow that marginal increases over standard dosing regimens will observably increase the likelihood of cure in any one individual. This error arises when individual cell survival curves are extrapolated into a single unified survival curve, and the assumption made that shifts along that composite cell survival curve will result in meaningful alterations in clinical outcomes for individuals. This represents a clear case of the ecological fallacy, or assuming that what is true of a group is also true of individual members of that group [7].

The issue of interindividual variability in tumor and normal tissue radiosensitivity was first quantified by a 1985 study of variability in radiation dose response within a set of patient-derived melanoma xenografts [8]. The fivefold variation in radiosensitivity observed among xenografts of the same histology but obtained from different patients was nearly the same as the degree of variability in radiosensitivity observed between “radioresistant” tumor cell lines (bladder cancer, non-small-cell lung cancer) and “radiosensitive” histologies (small-cell lung cancer, testicular teratoma). As the authors trenchantly noted, “histology is of little importance to predict the radiation response of tumours.” Contemporary studies, such as a recent radiosensitivity analysis in patient-derived colorectal cancer organoid cultures, have documented similar findings, with a “spectrum of radiosensitivities” encompassing an approximately 13-fold variability in D0 [9]. While it is appealing in theory to consider individualized radiosensitivity testing before beginning treatment in order to determine optimal doses for control, this would be a time- and resource-intensive process that is not readily translatable to current clinical practice. Since the radiosensitivity of a given tumor is not known before treatment, some patients receive more radiation than needed for cure, some could have been cured had a higher dose been given, and some cannot be cured without utilizing doses associated with a high risk of unacceptable toxicity.

It is, therefore, quite unlikely that the radiation dose received by any given patient is perfectly calibrated. (As an aside, this observation has additional implications for the recent trend of clinical research focused on non-inferiority trials comparing two different dosing regimens. If there is a range of doses likely to achieve similar tumor control, with testing of two essentially random doses along this continuum, the hypothesis that there is no difference between the two regimens is highly likely to be proven.) Yet, in the so-called “modern” radiation clinic, tumors of the same histology are all not only treated with the same radiation dose, but the precision RT paradigm also demands rigorous adherence to established dosing protocols, singularly focused on the technical requirements of delivering doses that are quite unlikely to actually represent the optimal treatment for any particular patient.

These practices are rooted in arguments made in the 1970s and 1980s, as RT protocols became increasingly formalized. Despite an acute awareness that not all tumors of the same histology will respond similarly to the same radiation dose, the dominant approach evolved to treat all patients, even in a population comprised of tumors with widely variable radiosensitivity, with the highest tolerable dose [10]. Compromise histology-specific dosing regimens were established at levels that were thought to optimize the balance between tumor control and treatment toxicity. Great vigilance was demanded against technical failures leading to a “marginal miss,” which, however, is only expected to be a problem if the “correct” dose is identified to begin with and the inflection point of the affected tumor’s dose-response curve lies precisely within the dose received within the fall-off region of the missed target. Additionally, if the dose in the fall-off region was truly critical to local control, techniques yielding a sharp penumbra such as proton therapy are expected to be associated with higher failure rates at the tumor periphery, which has not been observed.

Another purported benefit of increasing the anatomic precision of RT delivery is the reduction in toxicity risk associated with decreasing doses to neighboring OARs. The principle of “as low as reasonably achievable” (ALARA) is a fundamental tenet of radiation safety, and precision RT techniques typically reduce OAR doses. While some recent prospective work, especially in adaptive and MR-guided RT, has been confounded by the simultaneous deployment of adaptive techniques and target volume margin reduction, other studies of adaptive replanning, especially in the head and neck, have confirmed the ability of this technique to reduce the dose to the parotid glands and other critical structures [11-13]. Proton therapy can also substantially improve dose distribution in OARs near the target [14]. However, OAR dosing faces the same problem as tumor dosing: in most instances, the tolerance of OARs is not sufficiently well established to support the conjecture that modest reductions in specific dose metrics to OARs offered by advanced techniques will meaningfully decrease toxicity. Reinforcing this point, clinical experiences with MR-guided RT in pancreatic cancer [15], real-time adaptive RT in head and neck cancer [16], and proton therapy in prostate cancer [17,18] have not shown that OAR dose reductions afforded by these techniques significantly improve toxicity.

Evaluation of radiation dose-response effects in normal tissue is complicated by several factors, including methodological challenges arising from the fact that many severe RT toxicities are relatively rare and can occur months or years after treatment. Small single-institutional studies, which comprise the majority of normal tissue dose-response data in radiation oncology, are therefore unlikely to capture the true incidence of even moderately uncommon side effects. The probability (P) of a specified event not occurring in a study with sample size n is given by the formula P = (1 - p)^n^, where p is the true incidence of the event [19]. Consider a rare but serious radiation-induced side effect with a true incidence of 1%; a study with n of 50 will likely not observe this side effect (61% chance of no events) and even a 100-person study would only have a 63% chance of observing an event.

Serious statistical errors can additionally arise from the use of high-dimensional data collected from dose-volume histogram (DVH) curves to study infrequent events, further hampering our ability to establish normal tissue dose-response parameters with certainty [20]. As in malignant tissues, considerable interindividual variability in normal tissue radiosensitivity is well documented. One provocative analysis of radiation effects on parotid gland toxicity not only illustrates very high interindividual variability in RT-induced salivary gland damage risk but also suggests that there was no threshold dose below which loss of parotid gland function did not occur [21]. This observation, interestingly, is substantiated by one of the main arguments in favor of adaptive RT planning in the head and neck: shrinkage of the parotid gland beginning very early in the course of treatment, before the gland has received doses that are canonically believed to be “toxic.”

In summary, delivering RT with exquisite anatomic precision is only of clinical value if tumor dosing regimens are exactly correct, normal tissue constraints accurately reflect true dose-response relationships, and treatment planning has perfectly identified the target. However, it is clear that the present state of our knowledge is far from meeting these conditions. Failing to recognize the limits of our understanding of radiation dose-response effects in tissue risks enshrining flawed practices and preventing true advances in RT. Complacency regarding current standards of practice has been described as “medical Panglossianism,” or the belief that we are living in the “best of all possible worlds,” and is a serious obstacle to medical and scientific progress. One facet of medical Panglossianism is an emphasis on interventions that focus on altering arbitrary endpoints, thereby “treat[ing] doctors instead of patients” [22]. Precision RT approaches embody the Panglossian belief that optimal doses to the tumor and normal tissue have already been determined with near-complete accuracy, and the only challenge remaining is to ensure that these idealized doses are reproduced in the clinic with the utmost rigor. Such approaches treat our wildly inadequate understanding of cancer biology and radiation effects in tissue as settled science, constructing a fantasy of certainty in which current regimens are truly the best of all possible worlds. Unfortunately, this “precision” is illusory: technology cannot solve the biological problem of radioresistance. Over 40 years after Lester Peters remarked that “the fundamental problem of clinical radioresistance is…the inability to predictively identify its cause in the individual patient,” our field continues to struggle to surmount the core biological challenges of treating cancer with radiation, challenges that cannot be overcome by the use of ever more byzantine technological “solutions” to achieve marginal alterations in tumor dosing and OAR avoidance [23].

Practical considerations regarding the implementation of precision RT in the clinic

*"Do not squander time, for that is the stuff life is made out of."* -Benjamin Franklin, *Poor Richard's Almanack*

Setting theoretical critiques aside, there is only a limited set of circumstances where precision treatments are expected to yield even a dosimetric benefit compared with modern image-guided radiation therapy. In the case of real-time daily adaptive RT, there must be sufficient difference between fractions in the anatomic relationships of tumor and normal tissue to meaningfully affect dose distribution. Additionally, conventional methods such as diligent monitoring of on-board imaging findings and choice of appropriate planning target volume (PTV) margin must be unable to detect and account for these changes. Identifying patients who are most likely to benefit from this intensive intervention before beginning treatment is challenging despite efforts to describe patient and plan characteristics associated with a benefit to daily treatment adaptation [24]. While a discussion of patient selection criteria for proton therapy is beyond the scope of this paper, in most use cases, photon dose distributions fall well within acceptable parameters, and to date, studies of proton vs. photon therapy have either reported no difference in toxicity outcomes [17,18] or marginal changes in non-standard endpoints [25,26].

Stewardship of capital and human resources is also a question of great relevance to these expensive and time-intensive RT techniques. Healthcare systems are already plagued with issues of cost, access, and quality, not just in rural and other underserved areas. In the resource-limited environment that characterizes clinical research in radiation oncology, careful attention should be devoted to the question of how much time and money should be directed to developing ever more highly specialized treatments that are unlikely to be accessible to large segments of the population. Furthermore, even in the highest-resource settings, the precious commodity of Time is not an infinite one. Precision RT approaches must prove their ability to either improve disease control or reduce toxicity before the already strained capabilities of physicians and other clinical staff are further stretched to fulfill the time and resource demands of these approaches. An even more imperative need is high-quality research into mechanisms of radioresistance, interindividual variations in radiosensitivity, and other critical questions that affect radiation response in both malignant and normal tissues, in the hopes that a fuller understanding of radiation dose-response effects will someday allow radiation oncologists to truly claim the ability to deliver “precision” RT.
